# The Muscle Relaxation Effects of Gentle, Focal Load (4.9–7.4 N) With a Narrow Contact Area: A Narrative Review of Kanshoho and Conventional Manual Therapies

**DOI:** 10.7759/cureus.89646

**Published:** 2025-08-08

**Authors:** Takashi Sakato, Haruka Amitani

**Affiliations:** 1 Physiology (Fascia), Japan Health Organization, Chuo Ward, Tokyo, JPN; 2 Department of Psychosomatic Internal Medicine, Kagoshima University Graduate School of Medical and Dental Sciences, Kagoshima, JPN

**Keywords:** chronic low back pain, kanshoho, manual therapy, muscle stiffness, myofascial pain

## Abstract

In this narrative review, we summarize the immediate effects of Kanshoho, a novel manual therapy, in the context of chronic musculoskeletal pain. Excessive muscle stiffness is closely linked to chronic pain, highlighting the need for safe and rapid methods to relieve this tension. Kanshoho, a novel low-pressure muscle-relaxation technique developed in Japan in 2007, is characterized by the application of a very gentle, focal load (approximately 4.9-7.4  Newton (N), or ~500-750 gf) to a small area of the body’s surface while the patient performs guided active movements. This review summarizes the immediate effects of Kanshoho and identifies optimal application parameters from existing reports, and it compares Kanshoho’s outcomes with those of other common manual muscle-relaxation techniques.

In patients with chronic nonspecific low back pain, a brief Kanshoho session has been associated with immediate reductions in muscle stiffness and relief of pain. Furthermore, exploratory studies on technique parameters suggest that using a moderately gentle pressure (around 5-7 N) over a narrow contact area (~1-2 cm in diameter) yields the most pronounced muscle-relaxation effect. By contrast, substantially lower or higher pressures, or using a much broader contact area, appear less effective for achieving immediate stiffness reduction.

Compared to conventional manual therapies, such as massage, static stretching, proprioceptive neuromuscular facilitation, and myofascial release, Kanshoho produces similar or even greater improvements in muscle relaxation and pain relief, typically in a shorter amount of time and without causing post-treatment soreness. Its non-pharmacological and minimally invasive nature makes it especially suitable for elderly or hypersensitive individuals who might not tolerate more intense interventions. These findings highlight Kanshoho as a promising conservative approach for managing chronic myofascial pain (particularly nonspecific low back pain). However, further well-controlled studies are warranted to confirm its efficacy and to better understand the underlying mechanisms. As a narrative review, no formal statistical synthesis methods were applied.

## Introduction and background

Excessive muscle tension, often manifested as increased muscle stiffness, is considered a major contributor to chronic musculoskeletal pain such as low back pain (LBP) and shoulder stiffness. Recent systematic reviews highlight both the effectiveness and limitations of current treatments, such as physical activity and exercise, for chronic pain in adults, underscoring the ongoing need for minimally invasive interventions like Kanshoho [1]. Previous studies have reported that patients with chronic LBP exhibit significantly higher stiffness in the erector spinae and multifidus muscles compared to healthy controls, suggesting that reducing muscle stiffness may be an important therapeutic target in pain management [2].

Muscle stiffness is clinically defined as increased resistance of muscle tissue to deformation or stretch, often associated with pain or functional limitation. LBP refers to discomfort, tension, or stiffness localized between the lower ribs and the gluteal folds, frequently lacking identifiable structural abnormalities. Myofascial pain specifically describes pain originating from muscle tissue and associated fascia, commonly related to muscle stiffness or trigger points. Complementary and Alternative Medicine (CAM) encompasses healthcare practices used alongside or instead of conventional medicine, often characterized by holistic or integrative approaches.

LBP is highly prevalent, affecting approximately 10% of the Japanese population. It is the most commonly reported symptom among men and the second most common among women, following shoulder stiffness [3]. Globally, LBP remains a leading cause of disability, affecting hundreds of millions worldwide and resulting in substantial healthcare and economic burdens. However, in approximately 85% of cases, no specific structural abnormalities can be identified through imaging modalities such as X-rays or Magnetic Resonance Imaging (MRI), and these are categorized as non-specific LBP [4]. Due to this diagnostic ambiguity, determining effective treatment strategies remains challenging, and chronicity and recurrence are frequently observed.

As a result, in addition to standard therapies such as pharmacological treatment, spinal manipulation, and exercise therapy, various manual techniques have been widely employed to alleviate muscle tension. Conventional manual therapies commonly used for this purpose include massage (manual manipulation of soft tissues), static stretching (holding muscles at end-range positions to enhance flexibility), Proprioceptive Neuromuscular Facilitation (PNF; combining isometric contractions with assisted stretching), and myofascial release (applying sustained pressure to alleviate fascial adhesions). Many of these manual techniques are often derived from empirical or CAM approaches. In fact, many individuals with chronic LBP seek out CAM modalities such as massage and acupuncture [5], and LBP remains one of the most common indications for such therapies.

Among these alternative approaches, a novel manual technique called Kanshoho was developed in Japan in 2007 by Takashi Sakato. Initially regarded as a CAM‑based intervention, Kanshoho has gained increasing attention within the field of physical therapy in recent years and is now viewed as a neutral modality bridging both domains  [6-8]. The technique involves applying very gentle, focal pressure, typically 4.9-7.4 N, to the body surface over areas of muscle tension, while guiding the patient to perform repeated, low-amplitude, patient-initiated isotonic contractions and gentle active stretching of the target musculature at intervals of a few seconds [7]. In some cases, effective relaxation is obtained with loads as low as 0.98 N [6].

Each application affects a circular area of only ~1-2 cm in diameter, so treatment proceeds sequentially by gradually shifting the point of contact [7]. Owing to the extremely light load, Kanshoho is considered non‑invasive and painless, with virtually no risk of tissue damage [8]. Moreover, it requires only a few minutes per session to produce measurable muscle‑relaxation effects [7]. Since its development, Kanshoho has been adopted by patients and practitioners seeking relief from chronic pain conditions such as LBP, shoulder stiffness, and joint discomfort [8].

This review comprehensively summarizes the therapeutic procedures and physiological effects of Kanshoho, highlighting its efficacy, optimal application parameters, and safety profile. Furthermore, it evaluates its clinical utility through direct comparison with established manual muscle relaxation techniques commonly employed in physical therapy practice. To establish a solid conceptual foundation, we first provide an in-depth description of the therapeutic procedure and elucidate the distinctive biomechanical principles that underpin Kanshoho. The potential underlying mechanisms, such as mechanoreceptor activation, fascial fluid dynamics, and proprioceptive reflex modulation, will be discussed in detail in subsequent sections.

## Review

Methods

This narrative review summarizes key literature related to Kanshoho and comparative conventional manual therapies. The literature search was conducted using PubMed, Scopus, Web of Science, and the Cochrane Library, covering primarily studies published from 2007 (the year Kanshoho was first introduced) up to July 2025. Search terms included combinations of the following keywords: "Kanshoho," "manual therapy," "muscle stiffness," "low back pain," "fibromyalgia," "myofascial release," "massage," and "Proprioceptive Neuromuscular Facilitation (PNF)." Articles were selected based on their clinical relevance, methodological rigor, and their specific focus on gentle manual therapies for muscle relaxation. Specifically, we included original research articles (RCTs, clinical studies), case reports, and relevant systematic reviews and meta-analyses. Additionally, important foundational studies published before 2007 were selectively included to provide theoretical context and comparative benchmarks.

Screening Process

Literature selection was performed independently by two authors, who initially screened article titles and abstracts for relevance based on predefined inclusion and exclusion criteria. Full-text reviews were subsequently conducted to confirm eligibility. Although this is a narrative review and exact numbers are not reported, disagreements regarding inclusion were resolved through author discussion until consensus was reached.

Data Extraction Methods

Data extraction was performed qualitatively by two authors. Extracted information included study design, population characteristics, details of manual therapy interventions, biomechanical and neurophysiological mechanisms, key clinical outcomes (e.g., muscle stiffness, pain relief, range of motion), and treatment tolerability. Extracted data were compared and cross-checked between authors to ensure accuracy and consistency. Due to the narrative nature of this review, a formal risk-of-bias assessment was not conducted; however, the methodological quality of included studies was carefully considered. Extracted data were synthesized qualitatively, emphasizing comparative therapeutic outcomes and clarifying biomechanical and neurophysiological mechanisms. Although our review is narrative rather than systematic, we structured the search and reporting processes broadly following PRISMA guidelines, enhancing reproducibility and transparency of our methods.

Author Bias Acknowledgement

The authors acknowledge the potential for bias inherent in the narrative review format, particularly due to the primary author’s involvement in the development of Kanshoho. To mitigate this potential bias, literature selection and data extraction processes involved independent reviews by multiple authors, and care was taken to transparently describe comparative outcomes between Kanshoho and conventional manual therapies. Limitations of the included studies and the review process itself have been explicitly discussed.

Statistical Summary Method

Due to the narrative nature of this review, formal statistical methods such as meta-analysis or meta-regression were not performed. Accordingly, quantitative synthesis methods, including the calculation of p-values, confidence intervals (CIs), and effect sizes, were not utilized. Instead, findings from individual studies were qualitatively synthesized and summarized narratively. Individual studies included in this review sometimes reported statistical measures such as p-values or effect sizes. However, as our goal was narrative synthesis rather than quantitative integration, we reported these statistical measures only as presented in the original studies without further recalculations or statistical interpretation. The narrative approach thus provides qualitative context rather than quantitative statistical synthesis.

Therapeutic procedure of Kanshoho

Kanshoho is a manual therapy specifically developed to rapidly and non‑invasively reduce excessive muscle tension. A distinctive feature of Kanshoho is that the practitioner applies a light vertical load of 4.9-7.4 N over a small contact surface approximately 1 cm in diameter (≈ 0.8 cm²) while guiding the patient to perform two cycles of small, self‑initiated movements-such as lateral trunk flexion-at a tempo of roughly 4 seconds per back‑and‑forth cycle, followed by a pause of at least 2 seconds; this sequence is then repeated [7]. Load is applied using either a compressive rod or the practitioner’s fingertips and is maintained steadily throughout the procedure [7, 8]. For example, when treating the lower back, the patient is positioned sitting or standing and instructed to tilt the torso approximately 15° to each side, continuing this movement for about 5 to 10 minutes [7,8].

During the movement, the practitioner maintains a constant load without shifting the point of contact, ensuring uniform mechanical input regardless of muscle‑length changes caused by contraction and extension. Because each application affects only a limited area, roughly 1 cm in diameter, the practitioner gradually shifts the contact point to cover the full length of the targeted muscle through repeated applications [7]. This combination of extremely gentle mechanical stimulation and low-intensity repetitive movement has been shown to significantly reduce muscle stiffness [7]; however, the precise physiological mechanism, such as potential involvement of mechanoreceptors or fascial matrix remodeling, remains speculative and requires further investigation. Importantly, the technique seldom provokes pain during treatment, and the physical burden on the patient is minimal, making Kanshoho especially well-suited for elderly or physically frail individuals, a notable clinical advantage [7, 8].

Reduction in muscle stiffness and optimal load parameters

Recent studies have objectively demonstrated that Kanshoho can significantly reduce muscle stiffness within a very short time. In an early investigation by Sakaguchi et al., 15 adults with self‑reported chronic non‑specific LBP received a single 10‑minute session of Kanshoho applied unilaterally to the lumbar region. Pre‑/post‑treatment assessments showed a statistically significant decrease in lumbar muscle stiffness (p < 0.033), together with improvements in pain on a visual analogue scale (VAS) (p = 0.013) and in subjective sensations of bodily lightness and ease of movement [8].

Pre/post-treatment assessments showed a statistically significant decrease in lumbar muscle stiffness from 30.6 to 27.3 (mean reduction of approximately 11%, p < 0.001). The calculated effect size (Cohen’s d) for this reduction was approximately 0.47, indicating a moderate effect size. Improvements were also observed in pain assessed by VAS, decreasing significantly from 7.0 to 5.6 (approximately 20%, p = 0.013), as well as subjective sensations of bodily lightness and ease of movement (Table 1).

**Table 1:** Effects of a 10‑Minute Kanshoho intervention on lumbar muscle stiffness and subjective outcomes † VAS orientation converted: original Japanese article (0 = worst / 10 = best) → current table (0 = best / 10 = worst). Lower scores after intervention, therefore, indicate symptomatic improvement. Muscle hardness was measured using a digital muscle hardness meter (TDM-Z2 (BT)), which displays tissue stiffness on a numeric scale from 0 (equivalent to the softness of air) to 99 (equivalent to the hardness of metal).

Variable	Pre‑intervention (Mean ± SD)	Post‑intervention (Mean ± SD)	p‑value
Lumbar muscle stiffness (Measurement Point 3)	30.6 ± 6.7	27.3 ± 7.4	
LBP (VAS†)	7.0 ± 1.8	5.6 ± 2.0	
Body mobility (VAS†)	7.5 ± 1.9	4.6 ± 2.2	
Lightness of body (VAS†)	7.5 ± 1.9	4.4 ± 2.4	

To further investigate the optimal load parameters for Kanshoho, a subsequent study systematically examined variations in applied loads and contact-surface diameters. Sugimoto et al. treated 39 healthy participants under systematically varied applied loads (0, 2.5, 4.9, 7.4, and 9.8 N) and contact-surface diameters (ranging from 1 cm to 3 cm) to evaluate resulting changes in muscle stiffness [7]. The greatest reductions were observed with focal loads of 4.9 N and 7.4 N. In contrast, lighter loads (≤ 2.5 N) or heavier loads (≥ 9.8 N) were markedly less effective. Specifically, the post/pre stiffness ratios were 0.85 at 4.9 N and 0.87 at 7.4 N, compared with 0.95 at 2.5 N, 0.88 at 9.8 N, and 0.97 when no external load was applied (0 N), indicating virtually no change in the latter condition.

A similar pattern emerged for the contact area. A narrow diameter of 1-2 cm produced significant stiffness reductions, whereas a wider diameter of 3 cm produced little to no change; in some cases, stiffness even increased slightly (ratio = 1.06) [7]. These findings indicate that “moderate, focal loading”-specifically, 4.9-7.4 N applied through a 1-2 cm contact diameter-optimally promotes muscle relaxation. This applied load of 4.9-7.4 N is substantially lower than the forces typically reported for cervical mobilisation (22-92 N) [9] and considerably below the median force for Grade III lumbar mobilisation (164 N) [10] (Table 2). This uniquely gentle mechanical stimulus inherently ensures minimal invasiveness and superior safety, distinctly differentiating Kanshoho from conventional manual therapies. In the subsequent section, we comprehensively evaluate these safety characteristics and their generalizability, emphasizing their clinical significance across diverse patient populations.

**Table 2:** Effect of pressing intensity and contact area on muscle stiffness reduction Original values reported by Sugimoto et al. show the post/pre ratio of muscle hardness under each condition [7]. Percentage reduction is calculated here as (1 − post/pre ratio) × 100%. Positive values indicate a reduction in stiffness, whereas a negative value indicates a slight increase in stiffness. Notably, moderate pressing forces of 4.90 N–7.35 N (500–750 gf) with a small contact area (1–2 cm diameter) produced the greatest decrease in muscle stiffness.

Condition	Post/Pre Muscle Stiffness Ratio	Reduction in Muscle Stiffness (%)
Varying Pressing Force (contact area = 2 cm)		
No pressure (0 N)	0.97	3%
2.45 N (≈250 gf)	0.95	5%
4.90 N (≈500 gf)	0.85	15%
7.35 N (≈750 gf)	0.87	13%
9.80 N (≈1000 gf)	0.88	12%
Varying Contact Area (pressure = 4.90 N (≈500 gf))		
1 cm diameter	0.85	15%
2 cm diameter	0.87	13%
3 cm diameter	1.06	−6%

Generalizability and safety of the effects

The same study by Sugimoto et al. further examined whether individual factors-sex, Body Mass Index (BMI), and baseline muscle stiffness-affected the degree of stiffness reduction under focal loads of 4.9 N and 7.4 N. No statistically significant differences were found across these variables, suggesting that, with appropriate load adjustment, Kanshoho can elicit reproducible effects irrespective of sex, body habitus, or baseline stiffness [7]. For especially sensitive patients, such as those with fibromyalgia, starting with very light loads of ~0.98 N and gradually increasing the force has been reported to be effective [6]. No appreciable change in muscle stiffness was detected in untreated contralateral muscles, indicating that the primary effect of Kanshoho is localized to the site of mechanical input [7]. This focal selectivity strengthens the evidence for the technique’s safety profile.

Importantly, no participant reported muscle soreness or discomfort during or after treatment. Serum creatine‑kinase levels, an indicator of muscle damage, remained unchanged, and no adverse events were documented [8]. Taken together, these observations characterise Kanshoho as a minimally invasive intervention with an exceedingly low physical burden on patients.

This low invasiveness is especially valuable in clinical situations where conventional physical therapies are difficult to implement. For example, a 2024 case report by Amitani et al. used Kanshoho in a hospitalised patient with fibromyalgia and severe generalised allodynia [6]. Treatment began with an applied load of only 0.98 N and was progressively increased, as tolerated, to 1.96 N, 2.94 N, and finally 3.92 N. The treatment field was gradually expanded, starting at the occipital insertion of the trapezius and, over successive days, moving to the neck, shoulders, and back. After 20 days of daily sessions, the patient experienced a marked reduction in widespread pain [6].

This case, where even gentle touch had previously been intolerable, illustrates both the importance of muscle tension in chronic pain (especially fibromyalgia) and the safety and adaptability of Kanshoho. Moreover, the observation that localised treatment produced systemic pain relief hints at broader physiological effects, possibly mediated through fascial or neural networks. Taken together, current evidence indicates that Kanshoho, when delivered under its optimal loading parameters of 4.9-7.4 N applied through a 1-2 cm contact diameter, can safely and rapidly reduce muscle stiffness. Its benefits appear generalisable across diverse patient characteristics, including age, sex and body composition, positioning Kanshoho as a universally applicable, minimally invasive manual therapy.

Comparison with conventional muscle relaxation techniques

Massage Therapy

Massage is one of the most widely used manual therapies for alleviating chronic pain and reducing muscle tension, and it is frequently employed by both clinical populations and athletes [11-13]. The physiological mechanisms of massage therapy include mechanical stimulation that activates pressure receptors, potentially leading to increased vagal nerve activity and decreased substance P, a neurotransmitter associated with pain [12]. Additionally, massage has been shown to produce immediate, although short-term, reductions in muscle stiffness, directly contributing to improvements in flexibility and joint range of motion (ROM) [14].

Systematic reviews have confirmed that short-duration massage sessions can result in small but statistically significant improvements in flexibility and provide modest relief of delayed-onset muscle soreness (DOMS), suggesting benefits for recovery [13]. However, these positive effects are typically transient and diminish shortly after treatment [14]. Furthermore, the effectiveness of massage for substantial muscle relaxation may be limited, as many protocols involve relatively long session durations, and outcomes may vary according to the specific massage techniques used [12,13]. Some massage methods, especially those involving deeper pressure or sustained pressure on sensitive areas, can cause temporary discomfort or mild pain during treatment [14]. These factors represent notable limitations of massage therapy, highlighting considerations regarding patient comfort and the immediacy of therapeutic effects.

Static Stretching and PNF

Static stretching, which involves holding a muscle in an extended position for about 30 seconds, is known to reduce the sensitivity of muscle spindle reflexes and promote muscle relaxation as well as improve ROM. Budini et al. reported a significant reduction in stretch reflex activity and muscle stiffness immediately following static stretching, indicating a neurologically mediated decrease in spindle excitability [14].

PNF, on the other hand, is a manual stretching method based on post-isometric relaxation, commonly employing techniques such as Contract-Relax (CR) and Contract-Relax-Antagonist-Contract (CRAC). This technique involves voluntary isometric contraction of the target muscle followed by passive stretching, triggering autogenic inhibition via the Golgi tendon organ and reciprocal inhibition of the antagonist muscle. Hindle et al. concluded that PNF can effectively reduce muscle tension, alleviate pain, enhance ROM, and improve muscular performance [15].

Clinically, PNF is widely used for conditions such as chronic LBP and restricted ROM. However, it requires patients to exert muscular effort, posing challenges for those with severe pain, advanced age, or significant muscle weakness. Some guidelines even contraindicate PNF for frail or deconditioned individuals [16]. Additionally, a successful application requires precise resistance control and skilled guidance, leading to variability in outcomes depending on practitioner proficiency. Thus, while static stretching is broadly applicable and safe, PNF offers deeper effects under optimal conditions but has narrower indications and higher technical demands.

Myofascial Release (MFR)

Myofascial release (MFR) is a technique gaining increasing attention for its role in relieving fascial adhesions and restoring tissue mobility. It generally involves the application of low-load, sustained stretching or compression to the fascial complex to optimize tissue length, reduce pain, and improve function [17]. MFR has demonstrated effectiveness in treating chronic conditions such as myofascial pain syndrome caused by trigger points [18]. However, MFR typically requires several minutes of continuous pressure or traction, making immediate evaluation of effects difficult. Furthermore, in hypersensitive individuals, particularly patients with fibromyalgia syndrome (FMS), even mild stimulation can provoke discomfort, rendering traditional massage or MFR techniques impractical [5]. While some studies have shown improvements in tenderness and quality of life with appropriate MFR techniques [19], these findings underscore the importance of gentler approaches for highly sensitized patients.

In this context, Kanshoho has been reported to succeed even in refractory cases. It has been described as a uniquely gentle method suitable for severe FMS cases, where even slight stimuli can induce pain [5]. Some authors have suggested that Kanshoho may share mechanistic similarities with MFR, particularly in its potential to release fascial restrictions and improve the sliding of connective tissues [5]. However, Kanshoho differs in that it combines gentle mechanical input with active movement by the patient, and its underlying physiological mechanisms remain incompletely understood.

Some authors have suggested that Kanshoho may share mechanistic similarities with myofascial release (MFR), particularly in its potential to release fascial restrictions and improve sliding of connective tissues. However, Kanshoho differs by combining gentle mechanical input with patient-generated active movements. Table 3 summarizes these and other differences across various clinical dimensions, including mechanical load, patient activity, efficacy, pain during application, and skill requirements.

**Table 3:** Comparative features of Kanshoho and conventional muscle-relaxation techniques

Technique	Typical Mechanical Load	Patient Activity	Immediate Effect	Typical Duration (per region)	Pain / Discomfort During Application	Suitability for Pain Sensitive Cases (e.g. Fibromyalgia)	Practitioner Skill Requirement
Kanshoho	Very low point-load (~4.9–7.4 N [≈500–750 gf] on 1–2 cm²; ≤1.0 N [≈100 gf] in hypersensitive cases) [Present study, 5]	Active – small voluntary movements [5,7]	Yes – muscle stiffness falls within minutes [6]	~5–10 min [6]	None (painless) [7]	High – well tolerated, even in severe fibromyalgia [5]	Low–Moderate (based on expert consensus and clinical observation; basic training typically ensures high reproducibility among practitioners)
Massage (classical/deep tissue)	Low to moderate, therapist dependent [12,14]	Passive [12,14]	Yes – short-term relief of stiffness and improved flexibility [13,14]	~7–30 min [12,14]	Mild discomfort, particularly with deeper techniques [14]	Limited – short-term effects rapidly diminish; inconsistent effectiveness compared with active therapies [12,14]	Moderate (skill required to appropriately adjust pressure and optimize outcomes) [12,13]
Static Stretching	Tensile stretch (no external pressure) [14]	Passive [14]	Limited/modest – flexibility ↑, small acute stiffness ↓ [14]	2 × 30 s holds (≈1 min) [14]	Mild end range discomfort [14]	Good – gentle stretching usually accepted [14]	Low (simple to teach) [14]
Proprioceptive Neuromuscular Facilitation (PNF) Stretching (CR / CRAC)	Patient-generated strong isometric contraction followed by assisted stretch [15,16]	Active – contract–relax [15,16]	Yes – large, immediate ROM gain [15,16]	~1–2 min [15]	Moderate (intense effort/stretch) [15,16]	Poor – too intense for hypersensitive patients [15,16]	High (precise resistance & timing required) [15,16]
Myofascial Release (MFR)	Low–moderate sustained pressure, 1–3 min [17–19]	Passive [17–19]	Variable – release may take minutes, effect sometimes subtle [17,18]	1–3 min [17–19]	Mild–moderate; can be painful in oversensitised tissue [17,19]	Limited – only very gentle variants tolerated [17,19]	High (specialised fascia skills) [17]

Advantages of Kanshoho

Whereas conventional techniques such as massage, static stretching/PNF, and myofascial release all have therapeutic merit, each also carries specific limitations. Kanshoho, by contrast, achieves substantial muscle relaxation with extremely light, short-duration loading that does not provoke treatment pain. Muscle relaxation has been documented with loads as low as ~0.98 N (≈ 100 gf), although the most effective range appears to be 4.9-7.4 N [6]. When such light focal loads are combined with several minutes of active muscle contraction, intramuscular tension decreases markedly, thereby contributing to pain relief [5].

These beneficial effects are typically observed immediately following a brief Kanshoho session. Preliminary clinical reports indicate that symptomatic relief commonly persists for several hours up to a few days [5,7]. However, systematic studies investigating the precise duration of sustained relief are still limited, and further research is required to confirm these observations. For highly sensitised patients, such as those with fibromyalgia, treatment can begin with micro-loads of ~0.98 N and be increased incrementally as tolerated. Kanshoho is also extremely safe: its minimally invasive nature imposes negligible tissue stress, and the technique itself is simple enough that it does not demand advanced practitioner skill, thereby minimising inter-operator variability.

In summary, Kanshoho offers a rare combination of immediacy, safety, and procedural simplicity that sets it apart from traditional approaches. While it is not intended for conditions involving major structural pathology (e.g., fractures or tumours), it shows considerable promise as a conservative intervention for pain driven by muscular hypertonicity. Kanshoho may therefore constitute a valuable addition to the physical-therapy repertoire aimed at improving quality of life in patients with chronic musculoskeletal pain. Although Kanshoho is generally safe and suitable for a wide patient population, it is not recommended in cases involving acute inflammatory conditions, open wounds, skin lesions, or infections at the site of treatment. Practitioners should exercise caution and clinical judgment to avoid exacerbating local conditions or causing unnecessary discomfort.

Discussion

The introduction of Kanshoho has expanded the range of muscle relaxation approaches available for chronic pain rehabilitation. Although the technique has shown promising results in reducing muscle stiffness and alleviating pain, its precise physiological mechanisms remain incompletely understood [5].

Biomechanical Effects

One hypothesis suggests that Kanshoho exerts its primary effects biomechanically through the combination of sustained, gentle mechanical pressure and repetitive movement. This mechanical input may alter fluid distribution within the fascia and reduce fascial tension, thereby enhancing fascial mobility and directly resolving excessive muscle stiffness [8]. Prior studies have indicated that gentle and gradually increasing pressure can induce reorganization of adhesions and macromolecular structures within the fascia, restoring the mechanical properties and sliding capability of connective tissues [8]. In this context, Kanshoho may share mechanistic similarities with traditional myofascial release techniques, which similarly aim to address fascial restrictions through direct mechanical intervention.

Neurophysiological Effects

Another plausible mechanism involves neurophysiological responses triggered by the gentle pressure and low-amplitude repetitive movements characteristic of Kanshoho. These stimuli might directly influence mechanoreceptors, including muscle spindles and Golgi tendon organs [19]. Such sensory modulation could reduce muscle spindle firing rates, leading to reflexive decreases in muscle tone and tension [14,19]. The active movement component of Kanshoho, involving small voluntary muscle contractions by the patient, may significantly contribute to neuromodulation by stimulating large-diameter afferent fibers and activating central nervous system pain-gating mechanisms [20]. This central modulation could significantly contribute to pain relief observed clinically. However, these neurophysiological pathways require further targeted experimental investigation to confirm their precise role.

From a clinical perspective, a notable limitation of current research is that Kanshoho has primarily been investigated in relation to LBP and shoulder stiffness. Its effectiveness in other conditions, such as knee pain caused by lower limb hypertonicity or tension-type headache, has yet to be systematically explored. To clarify the scope of its indications, additional case reports and controlled trials targeting diverse conditions are needed. In particular, recent guidelines from EULAR (European Alliance of Associations for Rheumatology) indicate that exercise therapy is the only intervention receiving a 'strong for' recommendation, whereas conventional manual therapies were not granted such a recommendation [21]. Additionally, systematic reviews have suggested that manual therapy offers limited evidence of efficacy in fibromyalgia patients [22]. This context highlights the potential clinical advantage of Kanshoho, considering its demonstrated high tolerability and efficacy even in hypersensitive populations, such as fibromyalgia patients [6].

Establishing standardized protocols for Kanshoho is also crucial. Variables such as pressing force, duration, and type of movement (e.g., alternatives to lateral trunk flexion) should be optimized and unified across practitioners [6,23]. The lack of standardized procedures is a recognized issue in rehabilitation research broadly [23], and defining reproducible protocols for Kanshoho will improve the reliability of future outcome evaluations. Moreover, randomized controlled trials (RCTs) comparing Kanshoho directly with existing manual therapies, such as stretching and massage, represent an important next step. At present, the evidence supporting Kanshoho's efficacy is largely based on individual studies, which limits its strength. Well-designed RCTs evaluating metrics such as pain reduction, functional improvement, duration of effect, and safety profiles would help determine its relative utility. Given its noninvasive and low-burden nature, Kanshoho is well-suited to ethical and practical implementation in comparative trials.

**Figure 1:**
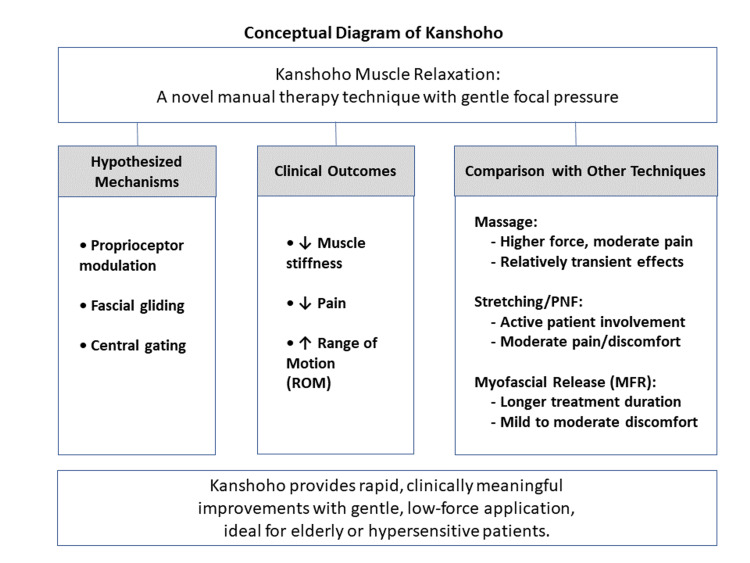
Conceptual diagram of Kanshoho Kanshoho's unique low-force approach promotes effective muscle relaxation through hypothesized mechanisms including proprioceptor modulation, fascial gliding, and central gating. Clinical outcomes such as reduced muscle stiffness, decreased pain, and improved ROM clearly distinguish it from conventional manual techniques, highlighting its particular suitability for elderly or hypersensitive populations.

As noted in recent systematic reviews, even widely accepted manual therapies like myofascial release lack high-level evidence for chronic musculoskeletal pain [24]. The establishment of Kanshoho as a validated technique will likewise depend on the accumulation of high-quality clinical research. To further illustrate Kanshoho’s clinical significance, Figure 1 presents a concise conceptual diagram summarizing its hypothesized mechanisms, key clinical outcomes, and clear comparisons with conventional manual therapies.

Emerging technologies such as shear wave elastography (SWE) have recently gained attention as reliable and quantitative methods for objectively assessing muscle stiffness [25]. Future research integrating Kanshoho with such advanced evaluation techniques would further validate its clinical effectiveness and provide robust physiological data to support its broader clinical application.

## Conclusions

This review has summarized Kanshoho, a novel manual technique characterized by gentle, focal loads (4.9-7.4 N) combined with patient-guided active movements. Kanshoho demonstrates rapid reductions in muscle stiffness and pain, typically within a brief treatment period, and offers distinct advantages such as minimal discomfort and suitability for elderly or hypersensitive individuals. While promising, existing evidence primarily originates from smaller-scale studies with limited conditions. Future research through large-scale, controlled trials directly comparing Kanshoho with conventional techniques is essential to establish broader clinical validation and fully understand its therapeutic potential. Clinically, Kanshoho represents a highly valuable and minimally invasive therapeutic addition to existing rehabilitation strategies, particularly for patients with chronic musculoskeletal pain who may not tolerate the higher-force or more invasive interventions of conventional manual therapies.
